# Advancing pearl millet yield forecasting: Comparative analysis of individual and ensemble machine learning approaches over Rajasthan, India

**DOI:** 10.1371/journal.pone.0317602

**Published:** 2025-03-11

**Authors:** Ahmad Alsaber, Parul Setiya, Anurag Satpathi, Abrar Aljamaan, Jiazhu Pan

**Affiliations:** 1 Department of Management, College of Business and Economics, American University of Kuwait, Salmiya, Kuwait; 2 Govind Ballabh Pant University of Agriculture and Technology, Pantnagar, Uttarakhand, India; 3 Division of Agrometeorology, Sher-e-Kashmir University of Agricultural Sciences and Technology, Kashmir Shalimar campus, Srinagar, India; 4 College of Art and Science, American University of Kuwait, Slamiya, Kuwait; 5 Department of Mathematics and Statistics, University of Strathclyde, Glasgow, United Kingdom; ICAR Central Coastal Agricultural Research Institute, INDIA

## Abstract

Pearl millet (*Pennisetum glaucum L.*) is a resilient crop known for its ability to thrive in arid and semi-arid regions, making it a crucial staple in regions prone to drought. Rajasthan, a state in India, emerged as the top producer of pearl millet. This study enhances yield forecasting for pearl millet using machine learning models across nine districts viz. Jaipur, Ajmer, Jodhpur, Bikaner, Bharatpur, Alwar, Sikar, Jhunjhunu and Nagaur in Rajasthan, India. Data from 1997–2019 (23 years), including yield data from the Directorate of Economics and Statistics and weather data from the NASA POWER web portal, were analysed. The study employed individual machine learning methods (GLM, ELNET, XGB, SVR and RF) and their ensemble combinations (GLM, ELNET, Cubist and RF). Discerning the overall best performing model across all locations remained challenging. For instance, while ensemble models exhibited subpar performance in Barmer and Nagaur, their performance ranged from satisfactory to commendable in other locations. To identify the best model, all models were ranked based on their R^2^ and nRMSE (%) values. Combined average ranks during training and testing revealed the model performance ranking as I-XGB (3.83) >  I-GLM (4.28) >  E-ELNET (4.32) >  I-RF (4.67) >  E-GLM (4.88) >  I-SVR (4.90) >  I-ELNET (4.94) >  E-RF (6.03) >  E-Cubist (7.15), where I denotes individual model, while E denotes ensemble model. Intriguingly, while individual GLM and XGB models demonstrated superior performance during calibration, they exhibited poorer performance during validation, potentially indicating issues of data overfitting. Hence, the ensemble ELNET approach is recommended for accurate prediction of pearl millet yield, followed by the individual RF model. These performances underscore the importance of tailored model selection based on specific geographic and environmental conditions.

## Introduction

Agriculture is a vital sector in India, playing a crucial role in ensuring food, nutrition, and livelihood security. It employs more than two-thirds of the workforce and significantly contributes to the country’s Gross Domestic Product (GDP) [1] Traditionally, Indian farming systems aimed to meet the dietary needs of both people and domestic animals, focusing on growing nutritious cereals such as millets and sorghum. However, with the commercialization of agriculture, farmers have shifted towards high-yield crops like rice and wheat. This change has led to increased malnutrition, undernourishment and micronutrient deficiencies. Therefore, there is a pressing need to adopt precision agriculture systems to enhance the yield and quality of highly nutritious crops other than main cereals.

In India, pearl millet (*Pennisetum glaucum L.*) is the fourth most widely cultivated food crop after rice, wheat, and maize [2]. It grows rapidly with minimal inputs, has high photosynthetic efficiency, and offers a balanced nutritional profile. It is also tolerant to extreme climatic conditions and biotic stresses. Recognizing its nutritional value, the Indian government has initiated a “millet revolution” to promote these grains as ‘Nutri Cereals’ for enhanced production, consumption, and trade [3]. Pearl millet is known for its resilience and low water requirements, has gained prominence. Its economic significance is clear from its contribution to exports and ongoing research aimed at enhancing its nutritional value and stress tolerance [4].

Pearl millet is a crucial crop in subsistence agriculture across the semi-arid tropics of India, where it is extensively cultivated for grain, fodder, and fuel [5,6]. It is the sixth most important cereal crop globally in terms of annual production [4,7]. Known locally as bajra in India, pearl millet significantly contributes to the agricultural landscape, generating approximately 20 million US dollars in millet exports during 2021–22. Its nutritional significance is underscored by its health benefits and potential use as an alternative poultry feed ingredient, demonstrating its versatility in addressing nutritional challenges [8].

Rajasthan, recognized as the “National Leader in Pearl Millets,” has the largest area and highest production of pearl millet in India. The state cultivates approximately 4.6 million hectares, with an average production of about 2.8 million tons and a productivity rate of 400 kg per hectare [8]. Rajasthan’s semi-arid climate, characterized by low and erratic rainfall, makes it a suitable region for cultivating drought-resistant crops like pearl millet. The significance of this study in this region lies in the fact that accurate yield predictions are crucial for policy planning, agricultural management, and food security initiatives. By focusing on Rajasthan, this study addresses the specific challenges faced by farmers in semi-arid regions, such as water scarcity and variable weather patterns. Accurate yield predictions can enhance resource allocation, improve food supply chain efficiencies, and inform decision-making at both governmental and farm levels. This, in turn, can help stabilize the livelihoods of farmers, optimize the use of agricultural inputs and contribute to the overall economic development of the region.

Moreover, monsoon systems, which play a critical role in facilitating agricultural activities across large parts of the world, have become increasingly unpredictable due to climate change. This unpredictability in rainfall and temperature gradients, as highlighted by [9], has direct implications for global and regional food security. Although pearl millet is known for its tolerance to abiotic and biotic stresses, the changing monsoonal patterns still pose challenges for its yield in regions like Rajasthan. Variations in precipitation and increased temperatures can negatively affect the growth cycle and yield outcomes, such variability leads to significant reductions in crop productivity. In light of these challenges, the use of machine learning models becomes even more critical. By analysing long-term weather patterns and yield data, the present study seeks to provide more accurate and location-specific forecasts that can help farmers in regions like Rajasthan adapt to the changing climatic conditions.

The arrival of machine learning and data analytics in agriculture presents new opportunities to tackle various challenges. Machine learning algorithms can analyse vast amounts of historical weather and crop yield data to identify patterns and make accurate predictions [10]. This technological advancement is particularly relevant for pearl millet, given its importance in regions like Rajasthan where traditional farming practices are heavily influenced by climatic variability. By integrating machine learning techniques, we can develop predictive models that not only forecast yields more accurately, but also provide actionable insights for farmers to optimize their practices in response to changing environmental conditions. Moreover, the comparative analysis of different machine learning models and their ensemble combinations represents a novel approach in the context of agricultural yield forecasting. While individual models have their strengths, ensemble methods, which combine multiple models, can often provide more robust and accurate predictions by leveraging the strengths of each component model [9–11].

Understanding climate behaviour and its effects on crop yields is essential for better yield prediction. Numerous studies have highlighted the significant impact of various weather variables on crop yield. Several researchers have developed pre-harvest yield forecasting models based on weather variables [12–14]. However, there is a need for more robust and accurate models that can provide reliable forecasts tailored to specific crops and regions. In light of this, our study aims to fill this gap by developing advanced machine learning models to predict the yield of pearl millet, utilizing 23 years of historical data from Rajasthan. The novelty of our research lies in the comparative analysis of individual machine learning methods (GLM, ELNET, XGB, SVR, and RF) and their ensemble combinations (GLM, ELNET, Cubist, and RF). By leveraging these advanced techniques, we seek to improve the accuracy and reliability of yield forecasts, which can significantly benefit farmers, policymakers, and stakeholders involved in agricultural planning and food security initiatives. This study not only contributes to the existing body of knowledge but also provides practical tools for enhancing agricultural productivity in semi-arid regions like Rajasthan.

## Materials and methods

### Data collection

**Fig 1:**
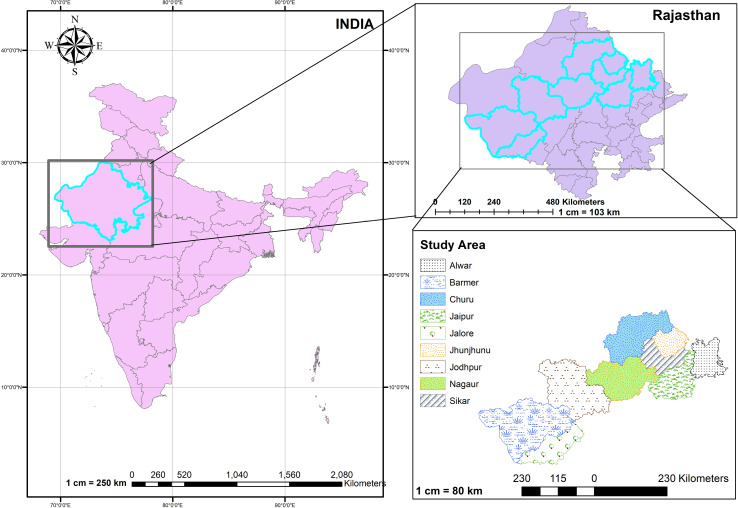
Study area, highlighting the nine major pearl millet-producing districts in Rajasthan.

To develop robust machine learning models for predicting pearl millet yield, we collected comprehensive data from multiple sources. Yield data spanning 23 years (1997–2019) were obtained from the Directorate of Economics and Statistics, Department of Agriculture and Farmers Welfare, Government of India, providing detailed records for nine major pearl millet-producing districts in the state. These districts include Jaipur, Ajmer, Jodhpur, Bikaner, Bharatpur, Alwar, Sikar, Jhunjhunu and Nagaur. Concurrently, weather data for the same period were gathered from the NASA Power website. This dataset encompassed crucial meteorological variables such as average weekly maximum temperature (°C), minimum temperature (°C), average relative humidity (%), wind speed (m/s), solar radiation and weekly accumulated rainfall (mm). The integration of these datasets allowed for a comprehensive analysis of the relationship between climatic conditions and pearl millet yields, forming the basis for our predictive modelling efforts. The meticulous collection and synchronization of yield and weather data were critical to ensuring the accuracy and reliability of our machine learning models. Fig 1 illustrates the area selected for this study.

### Weather indices

Two indices were developed for each weather variable: [1] an unweighted weather index, which is a simple sum of weekly weather variable values across different weeks, and [2] a weighted weather index, which is a total of these weekly values with weights derived from correlation coefficients. The weights are assigned based on the correlation coefficients between the target variable and the product of the weather variables for each week [15].

The computation of unweighted weather indices is straightforward and clear, involving a simple summation of weekly weather variable values without any weight. However, calculating weighted weather indices is a more intricate process that involves two main steps. First, correlation coefficients are calculated between the target variables and the weather variables for each week. This step establishes the relationship strength between each weather variable and the target variable. In the second step, the sum product is computed by multiplying each week’s weather variables by their respective correlation coefficients and then summing these products across the weeks. This process yields the weighted weather indices, which account for the varying influence of different weather variables on the target variable. This weighted approach ensures that more influential weather variables have a greater impact on the index, providing a more nuanced and accurate representation of weather impacts. This dual approach also allows for a more comprehensive analysis, accommodating the complexity and variability inherent in weather data and its effects on crop yield [16].

The weighted and unweighted weather indices were calculated using Equations (1) and (2)


$$Z_{ij}=\sum_{w=1}^{n}X_{iw}\;,\;Z_{ii'j}=\sum_{w=1}^{n}X_{iw}X_{i'w}$$ (1)

$$Z_{ij}=\sum_{w=1}^{n}r_{iw}^{j}X_{iw}\;,\;Z_{ii'j}=\sum_{w=1}^{n}r_{ii'w}^{j}X_{iw}X_{i'w}$$ (2)

Here, Z is the weather index of i^th^ weather variable, n is the week of forecast, $X_{iw}/X_{i'w}$ is the value of i^th^/i’^th^ weather variable and $r_{iw}^{j}/r_{ii'w}^{j}$ is the value of correlation coefficient of detrended yield with i^th^ weather variable/ product of i^th^ and i’^th^ weather variables in w^th^ week.

### Multivariate techniques involved in model development

In the present study, a variety of multivariate techniques were employed to develop a robust and accurate predictive model. These techniques enable the simultaneous analysis of multiple predictor variables, such as weather indices and historical yield data, to capture complex relationships and patterns in the data. Specifically, the individual methodologies utilized included Random Generalized Linear Models (GLM), Elastic Net (ELNET), Extreme Gradient Boosting (XGB), Support Vector Machines (SVR) and Random Forests (RF), to model the relationship between meteorological factors and pearl millet yield. Additionally, their ensemble approaches viz. GLM, ELNET, Cubist and RF were also utilized. It is noteworthy that 16 years of historical data were used for model training, while the remaining 7 years of data were reserved for model testing and validation, ensuring the robustness and generalizability of these predictive models.

### GLM

Generalized Linear Models (GLM) are highly effective tools for forecasting crop yields due to their flexibility in handling various types of response variables and their ability to model relationships between predictors and the response variable. GLMs extend linear models by allowing the response variable to follow different distributions from the exponential family, such as Gaussian, binomial, or Poisson, making them suitable for a range of agricultural data types. For crop yield forecasting, predictors can include environmental factors like temperature, precipitation, soil quality, and historical yield data. By incorporating these variables, GLMs can capture the complex interactions and non-linear relationships that influence crop productivity.

### Elastic net

Elastic Net, introduced by [17], addresses the limitations of ridge and LASSO regression methods. LASSO regression performs well with variables that are less correlated, while ridge regression is more effective with highly correlated variables. However, in models with numerous variables where the degree of correlation is unknown, both LASSO and ridge regression may fall short. Elastic Net overcomes this challenge by integrating the penalties of both LASSO (l1 norm) and ridge (l2 norm) regressions. This combination allows Elastic Net to accurately predict by accounting for both types of penalties.

### XG boost (XGB)

XGB is a highly scalable machine learning algorithm designed for tree boosting, as introduced by [18]. XGB is an ensemble method that leverages decision trees by building them sequentially, with each tree aiming to improve the performance of the previous one. The training process for each tree in XGB is parallelized, which significantly accelerates the overall training speed [19].

The core concept of XGB involves constructing multiple weak learners from the entire dataset and combining their outputs to enhance regression or classification performance. To mitigate overfitting, XGB employs a regularized model, where the weak learners can either be regression trees or linear models [18,20]. In this study, the XGB model is specifically built using decision trees. The algorithm generates predicted values by summing the leaf weights across all decision trees.

### Support vector regression

Support Vector Machine Regression (SVR) is a regression model derived from the Support Vector Machine (SVR) framework. Similar to SVR, SVR aims to find a regression plane that minimizes the distance of all input data points to this hyperplane. In essence, SVR necessitates the use of a kernel function to map inputs from the original space to a higher-dimensional space [21]. Subsequently, a linear function is established in this feature space to strike a balance between minimizing errors and preventing overfitting [22,23]. Commonly employed kernel functions in SVR encompass linear kernels, polynomial kernels, and Gaussian radial basis kernels. Moreover, the key hyperparameters that require tuning in SVR are the penalty coefficient C and the kernel coefficient gamma. SVR’s utilization of diverse kernel functions and hyperparameter tuning enables it to effectively model complex relationships in the data. By mapping inputs to higher-dimensional spaces and constructing linear functions, SVR can capture intricate patterns and deliver accurate regression predictions. The flexibility of SVR in handling various kernel functions and hyperparameters makes it a versatile and powerful tool for regression tasks across different domains.

### Random forest

The Random Forest (RF) regression algorithm is an ensemble-learning technique that integrates numerous regression trees to improve predictive accuracy. Each regression tree within the RF framework is structured as a series of hierarchical conditions, which are sequentially applied from the root to the terminal nodes, or leaves, of the tree [24–26]. When the Random Forest (RF) algorithm receives an input vector (x) containing the values of various evidential features analysed for a specific training area, it constructs a set of K regression trees and then averages their outcomes. Following the growth of K trees ${\left\{T\left(x\right)\right\}}_{1}^{k}$, the RF regression predictor can be expressed as the aggregation of the predictions from these trees [26].


$${\hat{f}}_{rf}^{k}\left(x\right)=\frac{1}{k}\overset{k}{\underset{k=1}{\sum }}T\left(x\right)$$ (3)

In order to mitigate the correlation among the individual trees within the Random Forest (RF) model, the algorithm enhances the diversity of the trees by having them develop from distinct training data subsets generated using a technique known as bagging [27]. Bagging is a technique aimed at reducing prediction variance to enhance a model’s generalizability by generating multiple trees from training data through sampling with replacement [28].

### Ensemble approach

In this study, ensemble approaches were also employed to enhance the predictive accuracy of machine learning models for pearl millet yield forecasting. Ensemble learning techniques combine multiple base models to improve overall performance by leveraging the strengths of individual models and mitigating their weaknesses. Specifically, four ensemble approaches were investigated: Generalized Linear Models (GLM), Elastic Net (ELNET), Cubist, and Random Forests (RF). These ensemble approaches integrate the predictions of multiple individual models, incorporating diverse perspectives and capturing a wider range of patterns in the data. By combining the predictions of individual models, ensemble approaches can often achieve higher predictive accuracy and robustness, making them well-suited for complex and dynamic agricultural systems like pearl millet cultivation.

### Evaluation of the model performance

To evaluate the performance of various models considered in the study, statistical measures such as the coefficient of determination (R²), root mean square error (RMSE) (t/ha), normalized root mean square error (nRMSE (%)), and Mean Biased Error (MBE) (t/ha) were calculated. A higher R² value closer to 1 and a lower RMSE value closer to 0 are indicative of better model performance. The nRMSE (%) values were classified as < 10% for excellent, 10–20% for good, 20–30% for fair, and >  30% for poor model performance [29]. These evaluation metrics were computed using the “apply Stats” function of R software, which in under “tdr’ library. These statistical measures are defined as:


$$R^{2}={\left(\frac{\frac{1}{n}\sum_{i=1}^{n}\left(y_{i}-\bar{y}\right)\left(\hat{y_{i}}-\bar{\hat{y}}\right)}{\sigma_{y}\sigma_{\hat{y}}}\right)}^{2}$$ (4)

$$RMSE=\sqrt{\frac{\sum_{i=1}^{n}{\left(y_{i}-{\hat{y}}_{i}\right)}^{2}}{n}}$$ (5)

$$MBE=\frac{1}{n}\sum_{i=1}^{n}\left(y_{i}-{\hat{y}}_{i}\right)$$ (6)

$$nRMSE=\sqrt{\frac{\sum_{i=1}^{n}{\left(y_{i}-{\hat{y}}_{i}\right)}^{2}}{n}}\times \frac{100}{\bar{A}}$$ (7)

Here $y_{i}$ represents the actual value and ${\hat{y}}_{i}$ represents the predicted value for i = 1, 2,……, n. $\sigma_{y}$ and $\sigma_{\hat{y}}$ represents the standard deviation of actual and predicted yield.

## Results

The descriptive statistics (Table 1) demonstrate the variability in the data used for model training and testing. For example, the yield values across the districts vary significantly, with a mean yield ranging from 0.25 to 1.65, and the sample variance in weather variables, such as temperature and rainfall, also shows substantial variability.

**Table 1:** Descriptive statistics of yield and weather parameters of different locations.

Parameter	Static parameter	Yield (t/ha)	Tmax (°C)	Tmin (°C)	Rain (mm)	RH (%)	WS (m/sec)	SR (W/m^2^)
Alwar	Mean	1.65	33.71	19.75	1.60	42.18	1.97	17.98
	Standard deviation	0.40	6.76	7.86	4.69	20.57	0.88	5.12
	Sample variance	0.16	45.71	61.82	22.02	422.98	0.77	26.19
Barmer	Mean	0.25	34.84	20.83	0.83	38.25	3.03	20.17
	Standard deviation	0.20	5.78	6.86	4.04	16.99	1.53	4.43
	Sample variance	0.04	33.47	47.05	16.33	288.95	2.34	19.66
Churu	Mean	0.44	33.41	20.21	1.19	36.95	2.16	19.31
	Standard deviation	0.26	7.11	7.91	3.93	16.51	0.99	5.09
	Sample variance	0.07	50.59	62.55	15.45	272.63	0.99	25.88
Jaipur	Mean	1.25	32.97	19.22	1.47	41.40	2.17	19.34
	Standard deviation	0.40	6.29	7.33	4.82	20.94	0.96	4.97
	Sample variance	0.16	39.66	53.79	23.21	438.40	0.92	24.73
Jalore	Mean	0.57	34.84	20.34	1.17	40.71	2.58	20.24
	Standard deviation	0.41	5.43	6.81	4.90	20.50	1.29	4.66
	Sample variance	0.17	29.45	46.44	24.02	420.31	1.68	21.76
Jhunjhunu	Mean	0.99	33.01	19.57	1.35	39.13	2.10	18.96
	Standard deviation	0.46	6.72	7.55	4.15	18.74	0.92	5.16
	Sample variance	0.21	45.18	56.99	17.22	351.17	0.85	26.67
Jodhpur	Mean	0.58	34.28	19.84	0.98	38.46	2.54	20.45
	Standard deviation	0.37	5.93	7.43	4.13	18.88	1.21	4.67
	Sample variance	0.14	35.20	55.19	17.09	356.32	1.47	21.86
Nagaur	Mean	0.78	33.81	19.59	1.04	38.41	2.45	19.98
	Standard deviation	0.33	6.22	7.50	3.60	18.61	1.15	4.85
	Sample variance	0.11	38.73	56.28	12.99	346.37	1.31	23.53
Sikar	Mean	0.97	32.87	19.43	1.32	39.12	2.25	19.18
	Standard deviation	0.37	6.50	7.36	4.23	18.63	0.98	5.03
	Sample variance	0.13	42.19	54.17	19.74	347.23	0.96	25.31

This table demonstrates significant variability in weather parameters such as temperature (Tmax and Tmin), rainfall, relative humidity, wind speed, and solar radiation, all of which contribute to the prediction of pearl millet yield using machine learning models.

The comprehensive results of our study, detailing the outcomes derived from the application of five individual machine learning models and four distinct ensemble modelling techniques across nine key pearl millet-producing districts are systematically discussed and described by the predictive performance of individual and ensemble models, offering insights into their effectiveness and reliability across the study regions.

### Individual models

**Fig 2:**
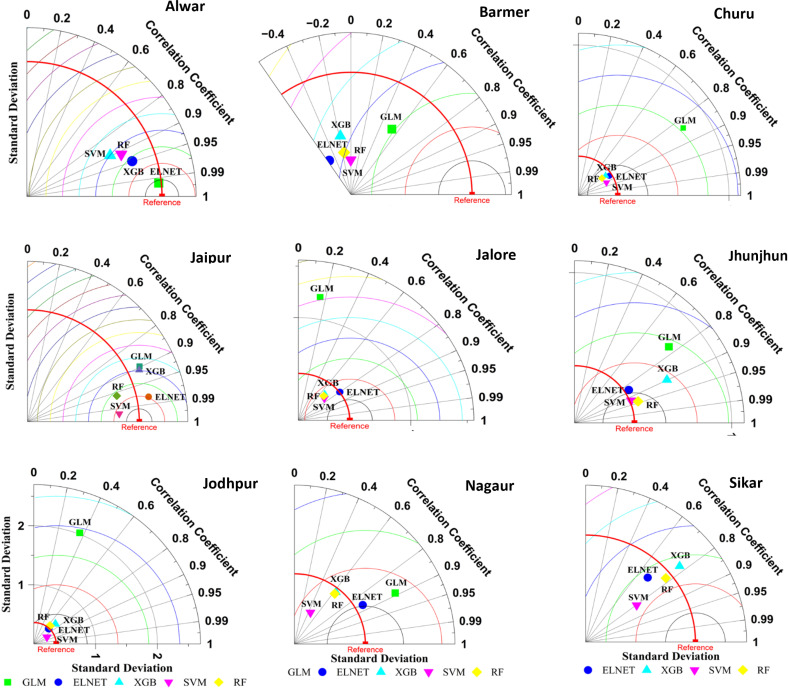
Taylor diagrams depicting the performance of individual models during validation.

Table 2 presents the performance metrics of individual models utilized for forecasting pearl millet yield, providing a comprehensive overview of their calibration and validation results. Fig 2 presents Taylor diagrams depicting the performance of individual models. The analysis of various individual models for predicting pearl millet yield in the Alwar district reveals noteworthy disparities in performance. During the calibration phase, all models exhibit commendable accuracy, with R-squared values ranging from 0.89 (ELNET) to 0.99 (GLM and XGB), alongside consistently low normalized Root Mean Square Error (nRMSE (%)) values, all below 0.16. Additionally, minimal Mean Biased Error (MBE) (t/ha) values near zero indicate the models’ proficiency in estimating pearl millet yield. However, upon validation, ELNET emerges as the top performer, boasting an impressive R-squared value of 0.99 and an associated nRMSE (%) of 0.13. Following closely is XGB with an R-squared value of 0.90 and nRMSE (%) of 0.15, indicating robust predictive capability. Notably, SVR and RF also demonstrate credible performance during validation, with R-squared values of 0.80 and 0.83 respectively, alongside relatively low nRMSE (%) values of 0.18 and 0.16, respectively. Conversely, GLM exhibits subpar performance during validation, with an R-squared value of 0.39 and a notably high nRMSE (%) value of 25.81. These findings emphasize the importance of careful model selection and validation in accurately forecasting pearl millet yield.

**Table 2:** Performance assessment of individual models during training and testing.

Alwar	Calibration					Validation				
	GLM	ELNET	XGB	SVR	RF	GLM	ELNET	XGB	SVR	RF
$R^{2}$	0.99	0.89	0.99	0.95	0.96	0.39	0.99	0.90	0.80	0.83
$RMSE\left(t/ha\right)$	0.00	0.13	0.00	0.13	0.11	24.79	0.12	0.15	0.17	0.15
$nRMSE\left(\%\right)$	0.00	0.09	0.00	0.09	0.16	25.81	0.13	0.15	0.18	0.16
$MBE\left(t/ha\right)$	0.00	0.00	0.00	0.02	0.00	−10.25	0.12	0.08	−0.01	−0.01
**Barmer**										
$R^{2}$	0.99	0.66	0.99	0.89	0.90	0.28	0.29	0.03	0.00	0.03
$RMSE\left(t/ha\right)$	0.00	0.12	0.00	0.09	0.07	0.14	0.19	0.19	0.16	0.17
$nRMSE\left(\%\right)$	0.00	0.19	0.00	0.14	0.10	0.32	0.46	0.44	0.38	0.41
$MBE\left(t/ha\right)$	0.00	0.00	0.00	−0.03	0.00	0.03	0.04	−0.02	−0.02	−0.01
**Churu**										
$R^{2}$	0.99	0.91	0.99	0.94	0.97	0.71	0.71	0.65	0.83	0.65
$RMSE\left(t/ha\right)$	0.00	0.08	0.00	0.08	0.06	0.58	0.13	0.17	0.12	0.15
$nRMSE\left(\%\right)$	0.00	0.09	0.00	0.09	0.07	0.96	0.21	0.28	0.20	0.24
$MBE\left(t/ha\right)$	0.00	0.00	0.00	0.01	0.01	0.21	−0.04	−0.10	−0.07	−0.05
**Jaipur**										
$R^{2}$	0.99	0.95	0.99	0.84	0.93	0.80	0.95	0.82	0.99	0.92
$RMSE\left(t/ha\right)$	0.00	0.09	0.00	0.17	0.13	0.20	0.19	0.18	0.08	0.13
$nRMSE\left(\%\right)$	0.00	0.06	0.00	0.11	0.08	0.20	0.19	0.17	0.08	0.11
$MBE\left(t/ha\right)$	0.00	0.00	0.00	0.00	0.00	−0.07	0.17	0.00	0.03	0.01
**Jalore**										
$R^{2}$	0.99	0.72	0.99	0.75	0.87	0.02	0.64	0.46	0.54	0.47
$RMSE\left(t/ha\right)$	0.00	0.22	0.01	0.23	0.17	1.53	0.28	0.30	0.29	0.29
$nRMSE\left(\%\right)$	0.00	0.15	0.00	0.15	0.12	1.43	0.26	0.28	0.27	0.27
$MBE\left(t/ha\right)$	0.00	0.00	0.00	−0.03	0.00	−1.09	0.12	0.08	0.10	0.01
**Jhunjhunu**										
$R^{2}$	0.99	0.95	0.99	0.95	0.96	0.58	0.71	0.81	0.85	0.89
$RMSE\left(t/ha\right)$	0.00	0.10	0.04	0.12	0.10	0.74	0.23	0.34	0.13	0.14
$nRMSE\left(\%\right)$	0.00	0.06	0.02	0.07	0.06	0.80	0.25	0.37	0.14	0.15
$MBE\left(t/ha\right)$	0.00	0.00	0.00	−0.03	0.00	0.57	0.13	0.16	0.03	0.07
**Jodhpur**										
$R^{2}$	0.99	0.78	0.99	0.83	0.96	0.13	0.47	0.52	0.77	0.42
$RMSE\left(t/ha\right)$	0.00	0.18	0.00	0.20	0.09	1.90	0.28	0.30	0.22	0.29
$nRMSE\left(\%\right)$	0.00	0.16	0.00	0.17	0.08	2.18	0.32	0.35	0.26	0.33
$MBE\left(t/ha\right)$	0.00	0.00	0.00	−0.06	0.00	0.92	−0.13	−0.07	−0.15	−0.09
**Nagaur**										
$R^{2}$	0.99	0.80	0.99	0.86	0.96	0.79	0.75	0.39	0.20	0.39
$RMSE\left(t/ha\right)$	0.00	0.15	0.02	0.14	0.08	0.31	0.18	0.27	0.29	0.27
$nRMSE\left(\%\right)$	0.00	0.13	0.02	0.12	0.07	0.36	0.21	0.32	0.34	0.32
$MBE\left(t/ha\right)$	0.00	0.00	0.00	−0.04	0.00	−0.16	−0.02	−0.01	0.05	0.05
**Sikar**										
$R^{2}$	0.99	0.85	0.99	0.78	0.92	0.49	0.47	0.59	0.65	0.60
$RMSE\left(t/ha\right)$	0.00	0.15	0.01	0.20	0.12	0.57	0.24	0.24	0.20	0.22
$nRMSE\left(\%\right)$	0.00	0.12	0.01	0.16	0.09	0.64	0.27	0.27	0.23	0.25
$MBE\left(t/ha\right)$	0.00	0.00	0.00	0.00	0.01	−0.18	−0.05	0.06	0.01	0.08

For Barmer district, during the calibration phase, models like GLM, XGB and RF demonstrated high accuracy, with R-squared values ranging from 0.90 (RF) to 0.99 (GLM and XGB). However, ELNET (R^2^ =  0.66) and SVR (R^2^ =  0.89) exhibited slightly lower performance in comparison. Notably, all models displayed minimal Root Mean Square Error (RMSE) (t/ha) values during calibration, indicating precise forecasting. However, during validation, the predictive accuracy decreased for all models possibly due to overfitting of the data, with R-squared values ranging from 0.00 (SVR) to 0.29 (ELNET). ELNET (R^2^ =  0.29) and GLM (R^2^ =  0.28) maintained relatively higher R-squared values compared to other models. Nevertheless, all models showed an increase in RMSE (t/ha) and normalized RMSE (nRMSE (%)) values during validation, suggesting a decrease in predictive accuracy. Overall, while certain individual models performed well during calibration, their predictive power diminished during validation, highlighting the complexity of accurately forecasting pearl millet yield for the Barmer district.

For the Churu district, during the calibration phase, all models demonstrated high accuracy, with R-squared values ranging from 0.91 to 0.99. Particularly noteworthy is the performance of GLM, XGB and RF, which exhibited exceptionally high R-squared values of 0.99, 0.99 and 0.97, respectively, indicating strong predictive capability. Minimal Root Mean Square Error (RMSE) (t/ha) values across all models during calibration further support their precise forecasting accuracy. However, during validation, there was a noticeable decrease in predictive accuracy for all models, with R-squared values ranging from 0.65 to 0.83. Despite this decline, SVR demonstrated the highest R-squared value of 0.83 during validation, indicating relatively robust predictive performance. Nevertheless, an increase in RMSE (t/ha) and normalized RMSE (nRMSE (%)) values during validation suggests a decrease in overall predictive accuracy compared to the calibration phase. Additionally, while most models displayed minimal Mean Biased Error (MBE) (t/ha) values close to zero during calibration, slight discrepancies were observed during validation, indicating some level of bias in the predicted values.

For the Jaipur district, during the calibration phase, all models exhibited high accuracy, with R-squared values ranging from 0.84 to 0.99. Particularly noteworthy is the performance of GLM and XGB, which demonstrated R-squared values of 0.99, indicating strong predictive capability. Additionally, all models displayed minimal Root Mean Square Error (RMSE) (t/ha) and normalized RMSE (nRMSE (%)) values during calibration, indicative of precise forecasting accuracy. During validation, the predictive performance of the models remained relatively strong, with R-squared values ranging from 0.80 to 0.99. SVR stood out with the highest R-squared value of 0.99 during validation, highlighting its robust predictive accuracy. Notably, while most models maintained minimal RMSE (t/ha) and nRMSE (%) values during validation, slight variations were observed. For instance, GLM, ELNET and XGB exhibited slightly higher RMSE (t/ha) and nRMSE (%) values compared to other two models. Moreover, despite minor discrepancies, all models displayed minimal Mean Biased Error (MBE) (t/ha) values close to zero during validation, indicating overall unbiased predictions. Overall, the results suggest that various models can provide accurate forecasts of pearl millet yield for the Jaipur district.

For the Jaipur district, during the calibration phase, all models exhibited high accuracy, with R-squared values ranging from 0.84 to 0.99. Particularly noteworthy is the performance of GLM and XGB, which demonstrated R-squared values of 0.99, indicating strong predictive capability. Additionally, all models displayed minimal Root Mean Square Error (RMSE) and normalized RMSE (nRMSE (%)) values during calibration, indicative of precise forecasting accuracy. During validation, the predictive performance of the models remained relatively strong, with R-squared values ranging from 0.80 to 0.99. SVR stood out with the highest R-squared value of 0.99 during validation, highlighting its robust predictive accuracy. Notably, while most models maintained minimal RMSE (t/ha) and nRMSE (%) values during validation, slight variations were observed. For instance, GLM, ELNET and XGB exhibited slightly higher RMSE (t/ha) and nRMSE (%) values compared to other two models. Moreover, despite minor discrepancies, all models displayed minimal Mean Biased Error (MBE) (t/ha) values close to zero during validation, indicating overall unbiased predictions. Overall, the results suggest that various models can provide accurate forecasts of pearl millet yield for the Jaipur district.

For the Jalore district, during the calibration phase, notable variations in performance are observed among the models, with GLM and XGB demonstrating high accuracy, reflected in R-squared values of 0.99. Conversely, ELNET and SVR show comparatively lower R-squared values of 0.72 and 0.75, respectively during calibration. Despite this, all models display minimal Root Mean Square Error (RMSE) (t/ha) and normalized RMSE (nRMSE (%)) values, indicating precise forecasting accuracy during calibration. However, during validation, the predictive performance of the models diminishes, with R-squared values ranging from 0.02 to 0.64. Notably, GLM performs poorly during validation, with an R-squared value of 0.02, indicating limited predictive capability. ELNET stands out with the highest R-squared value of 0.64 during validation, demonstrating relatively stronger predictive accuracy compared to other models. Nevertheless, all models exhibit an increase in RMSE (t/ha) and nRMSE (%) values during validation, suggesting a decrease in overall predictive accuracy compared to the calibration phase. Additionally, while most models display minimal Mean Biased Error (MBE) (t/ha) values close to zero during validation, GLM exhibits a substantial negative MBE (t/ha) value, indicating a bias towards underestimation in its predicted values.

For the Jhunjhunu district, during the calibration phase, all models demonstrate high accuracy, with R-squared values ranging from 0.95 to 0.99. Notably, GLM and XGB models exhibit particularly strong performance, with R-squared values of 0.99, indicating robust predictive capabilities. Additionally, all models display minimal Root Mean Square Error (RMSE) (t/ha) and normalized RMSE (nRMSE (%)) values during calibration, suggesting precise forecasting accuracy. However, during validation, the predictive performance of the models varies. While GLM and XGB models maintain a good R-squared values of 0.58 and 0.71 respectively, SVR, RF, and ELNET models demonstrate even higher R-squared values, ranging from 0.81 to 0.89. Notably, RF and SVR models exhibit the highest R-squared values during validation, suggesting strong predictive accuracy. Nevertheless, all models experience an increase in RMSE (t/ha) and nRMSE (%) values during validation, indicating a decrease in overall predictive accuracy compared to the calibration phase. Positive MBE (t/ha) values during the validation stage also suggest overestimation of the pearl millet yield.

For the Nagaur district, during the calibration phase, all models exhibit varying degrees of accuracy, with R-squared values ranging from 0.80 to 0.99. Notably, GLM and XGB models demonstrate particularly strong performance, boasting R-squared values of 0.99, indicative of their robust predictive capabilities. Moreover, all models demonstrate minimal Root Mean Square Error (RMSE) (t/ha) and normalized RMSE (nRMSE (%)) values during calibration, indicating precise forecasting accuracy. However, during validation, the predictive performance of the models varies. While GLM (0.79) and ELNET (0.75) models maintain relatively high R-squared values during validation, XGB (0.39), SVR (0.20) and RF (0.39) models show comparatively lower R-squared values. Nonetheless, all models experience an increase in RMSE (t/ha) and nRMSE (%) values during validation, indicating a decrease in overall predictive accuracy compared to the calibration phase. Notably, several models exhibit biases in their predicted values during validation, as indicated by the Mean Biased Error (MBE) (t/ha) values deviating from zero.

For the Sikar district, during the calibration phase, all models demonstrate varying levels of accuracy, with R-squared values ranging from 0.78 to 0.99. Notably, GLM and XGB models exhibit particularly strong performance, achieving R-squared values of 0.99, indicative of robust predictive capabilities. Additionally, all models display minimal Root Mean Square Error (RMSE) (t/ha) and normalized RMSE (nRMSE (%)) values during calibration, suggesting precise forecasting accuracy. However, during validation, the predictive performance of the models varies. While GLM and ELNET models maintain relatively low R-squared values of 0.49 and 0.47 during validation, SVR, RF and XGB models show comparatively higher R-squared values of 0.65, 0.60 and 0.59, respectively. Nonetheless, all models experience an increase in RMSE (t/ha) and nRMSE (%) values during validation, indicating a decrease in overall predictive accuracy compared to the calibration phase. It’s noteworthy that all models exhibit biases in their predicted values during validation, as indicated by Mean Biased Error (MBE) (t/ha) values deviating from zero except SVR (MBE =  0.01).

### Ensemble models

**Fig 3:**
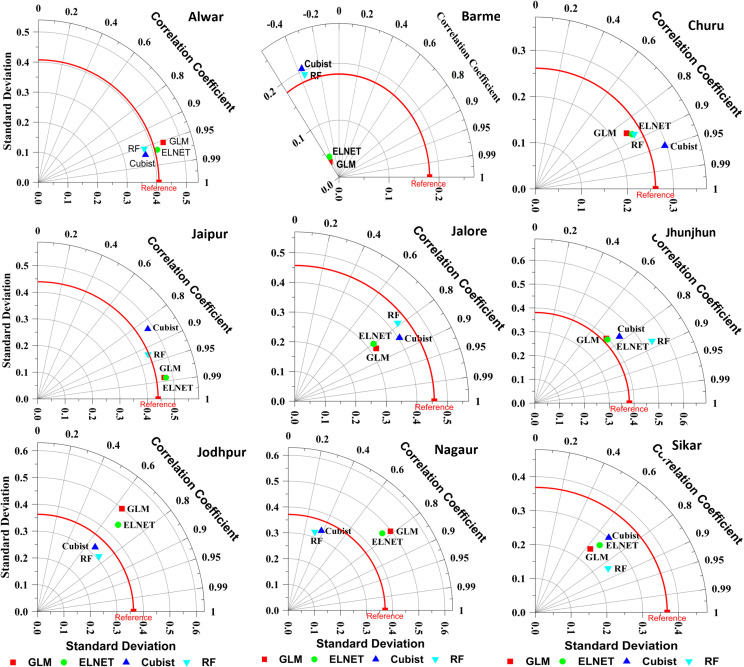
Taylor diagrams representing the performance of ensemble models during validation.

Ensemble approaches were offering their efficacy throughout both calibration and validation stages (Table 3). Fig 3 represents the Taylor diagrams representing the performance of ensemble models. For Alwar district, while maintaining a similar pattern to individual models, the ensemble models exhibit diverse levels of accuracy during calibration, with R-squared values ranging from 0.82 (Cubist) to 0.98 (GLM and ELNET). Notably, all ensemble models demonstrate minimal Mean Biased Error (MBE) (t/ha) values, all negative but very close to zero, indicating a slight underperformance. Upon validation, the ensemble models uphold their predictive ability, with R-squared values varying from 0.91 to 0.94. Of particular interest is the consistent performance of Random Forest (RF) with an R-squared value of 0.91 and the robust performance of Cubist with an R-squared value of 0.94. A comparative analysis reveals that while individual models XGB and ELNET showcased superior performance, in the ensemble approach, ELNET emerged as the top performer. However, upon holistic comparison across all models, XGB maintains its position as the best-performing model, with a slight margin evident in the statistical metrics.

**Table 3:** Performance assessment of ensemble models during training and testing.

Alwar	Calibration				Validation			
	GLM	ELNET	SVR	RF	GLM	ELNET	XGB	SVR
$R^{2}$	0.98	0.98	0.82	0.86	0.91	0.93	0.94	0.91
$RMSE\left(t/ha\right)$	0.07	0.06	0.20	0.16	0.13	0.12	0.13	0.10
$nRMSE\left(\%\right)$	0.05	0.04	0.14	0.11	0.14	0.12	0.13	0.11
$MBE\left(t/ha\right)$	−0.01	−0.01	−0.01	−0.05	0.06	0.07	−0.09	−0.01
**Barmer**								
$R^{2}$	0.44	0.70	0.37	0.39	0.31	0.22	0.13	0.13
$RMSE\left(t/ha\right)$	0.15	0.13	0.17	0.16	0.17	0.18	0.29	0.27
$nRMSE\left(\%\right)$	0.28	0.20	0.26	0.25	0.41	0.41	0.67	0.63
$MBE\left(t/ha\right)$	−0.01	−0.01	0.03	−0.01	0.01	0.01	0.08	0.04
**Churu**								
$R^{2}$	0.95	0.95	0.68	0.76	0.73	0.76	0.90	0.77
$RMSE\left(t/ha\right)$	0.06	0.06	0.15	0.13	0.13	0.12	0.08	0.12
$nRMSE\left(\%\right)$	0.07	0.07	0.18	0.15	0.22	0.20	0.14	0.20
$MBE\left(t/ha\right)$	−0.01	−0.01	−0.03	−0.01	−0.06	−0.05	0.02	−0.04
**Jaipur**								
$R^{2}$	0.96	0.96	0.87	0.96	0.97	0.97	0.70	0.85
$RMSE\left(t/ha\right)$	0.08	0.08	0.14	0.09	0.15	0.15	0.28	0.15
$nRMSE\left(\%\right)$	0.06	0.05	0.10	0.06	0.15	0.14	0.27	0.15
$MBE\left(t/ha\right)$	−0.02	−0.01	−0.03	−0.03	0.13	0.13	0.16	0.02
**Jalore**								
$R^{2}$	0.72	0.72	0.30	0.72	0.69	0.64	0.72	0.62
$RMSE\left(t/ha\right)$	0.27	0.26	0.35	0.30	0.24	0.25	0.33	0.30
$nRMSE\left(\%\right)$	0.18	0.17	0.23	0.28	0.22	0.23	0.31	0.28
$MBE\left(t/ha\right)$	−0.06	−0.02	0.09	0.17	−0.08	0.06	0.25	0.17
**Jhunjhunu**								
$R^{2}$	0.95	0.96	0.89	0.92	0.53	0.54	0.60	0.77
$RMSE\left(t/ha\right)$	0.13	0.13	0.18	0.14	0.31	0.30	0.35	0.31
$nRMSE\left(\%\right)$	0.08	0.08	0.11	0.08	0.33	0.32	0.37	0.33
$MBE\left(t/ha\right)$	0.01	0.01	0.01	−0.03	0.18	0.18	0.25	0.19
**Jodhpur**								
$R^{2}$	0.98	0.96	0.78	0.76	0.41	0.47	0.45	0.56
$RMSE\left(t/ha\right)$	0.06	0.07	0.20	0.18	0.35	0.30	0.29	0.31
$nRMSE\left(\%\right)$	0.05	0.06	0.18	0.16	0.40	0.34	0.33	0.36
$MBE\left(t/ha\right)$	0.00	0.00	0.05	−0.01	−0.09	−0.09	−0.15	−0.23
**Nagaur**								
$R^{2}$	0.95	0.97	0.67	0.80	0.62	0.59	0.14	0.10
$RMSE\left(t/ha\right)$	0.09	0.08	0.18	0.14	0.27	0.26	0.34	0.35
$nRMSE\left(\%\right)$	0.08	0.07	0.16	0.13	0.31	0.30	0.40	0.41
$MBE\left(t/ha\right)$	0.05	0.05	0.01	0.00	−0.04	−0.03	0.04	0.03
**Sikar**								
$R^{2}$	0.49	0.83	0.51	0.70	0.78	0.45	0.46	0.71
$RMSE\left(t/ha\right)$	0.57	0.16	0.27	0.20	0.18	0.25	0.26	0.19
$nRMSE\left(\%\right)$	0.64	0.13	0.22	0.16	0.14	0.28	0.29	0.21
$MBE\left(t/ha\right)$	−0.18	−0.01	0.01	0.02	−0.01	−0.08	−0.11	−0.05

In case of ensemble approaches, the combined models showed varying levels of accuracy for Alwar district, with R-squared values ranging from 0.37 (Cubist) to 0.70 (ELNET) during the training stage. ELNET demonstrated the highest R-squared value of 0.70 among the combined models. However, during validation, the predictive accuracy decreased again for all combined models, with R-squared values ranging from 0.13 to 0.31. GLM and ELNET displayed the highest R-squared values during validation, which is 0.31 and 0.22, respectively. Nonetheless, all combined models exhibited an increase in Root Mean Square Error (RMSE) (t/ha) and normalized RMSE (nRMSE (%)) values during validation, indicating a decrease in predictive accuracy compared to the calibration phase. Additionally, positive MBE (t/ha) values of all ensemble approach also suggesting overestimation of the yield. The overall comparison of the individual and ensemble models shows ELNET is the best approach in both scenarios. However, ensemble model like ELNET, showcased improved predictive accuracy during validation compared to individual models. Despite this improvement, both individual and ensemble models experienced a decrease in predictive accuracy during validation, as indicated by higher RMSE (t/ha) and nRMSE (%) values.

For Churu district, during the calibration phase, the ensemble models exhibited varying degrees of accuracy, with R-squared values ranging from 0.68 to 0.95. Notably, ELNET and GLM ensemble models displayed robust performance, demonstrating high R-squared values of 0.95 for both. However, Cubist and RF ensemble models showed comparatively lower R-squared values, indicating relatively weaker predictive capabilities during calibration. Despite these variations, all ensemble models demonstrated minimal Root Mean Square Error (RMSE) (t/ha) values, indicative of precise forecasting accuracy during calibration. During validation, the ensemble models maintained their predictive prowess, with R-squared values ranging from 0.73 to 0.90. Notably, Cubist ensemble models showcased the highest R-squared value of 0.90, indicating strong predictive performance during validation. Conversely, RF, ELNET and GLM ensemble models also demonstrated commendable performance, with R-squared values of 0.77, 0.76 and 0.73, respectively. Overall, based on the R-squared values of validation phase the SVR individual model stands out as the top performer among individual models, while the Cubist ensemble model exhibits the highest accuracy among the ensemble models.

For Jaipur, during the calibration phase, the ensemble models exhibited varying degrees of accuracy, with R-squared values ranging from 0.87 to 0.96. Notably, GLM, ELNET and RF ensemble models displayed robust performance, demonstrating high R-squared values of 0.96. However, Cubist ensemble models showed comparatively lower R-squared values, indicating relatively weaker predictive capabilities during calibration. Despite these variations, all ensemble models demonstrated minimal Root Mean Square Error (RMSE) (t/ha) and normalized RMSE (nRMSE (%)) values, indicative of precise forecasting accuracy during calibration. During validation, the ensemble models maintained their predictive prowess, with R-squared values ranging from 0.70 to 0.97. Notably, GLM and ELNET ensemble models exhibited the highest R-squared values of 0.97, indicating strong predictive performance during validation. Conversely, Cubist ensemble models showed a lower R-squared value, suggesting comparatively weaker predictive capabilities. Despite the overall strong performance of ensemble models, it’s worth noting that these models exhibited slightly higher Mean Biased Error (MBE) (t/ha) values during validation, indicating overestimation in the predicted values except RF. Nonetheless, the consistent accuracy demonstrated by the ensemble models highlights their effectiveness in predicting pearl millet yield for the Jaipur district, underscoring their relevance in informing agricultural decision-making processes.

For Jalore, during the calibration phase, the ensemble models exhibit varying degrees of accuracy, with R-squared values ranging from 0.30 to 0.72. Notably, ELNET and RF ensemble models demonstrate relatively higher R-squared values of 0.72, indicating stronger predictive capabilities. However, Cubist ensemble models display a comparatively lower R-squared value of 0.30 during calibration. Despite these differences, all ensemble models show similar Root Mean Square Error (RMSE) (t/ha) and normalized RMSE (nRMSE (%)) values, suggesting comparable forecasting accuracy during calibration. During validation, the predictive performance of the ensemble models remains consistent, with R-squared values ranging from 0.62 to 0.72. Cubist ensemble models demonstrate the highest R-squared value of 0.72 during validation, indicating robust predictive accuracy. Conversely, RF ensemble models exhibit a slightly lower R-squared value of 0.62, suggesting comparatively weaker predictive capabilities. However, all ensemble models maintain minimal RMSE (t/ha) and nRMSE (%) values during validation, indicative of accurate predictions. While most ensemble models exhibit minimal Mean Biased Error (MBE) (t/ha) values close to zero during validation, Cubist and RF ensemble models display a higher MBE (t/ha) value, suggesting a potential bias in its predicted values. Overall, the ensemble models cannot outperform the individual models during the calibration stage, while during the validation the ensemble models outperform all individual models for pearl millet production over Jalore district.

For Jhunjhunu, during the calibration phase, all ensemble models demonstrate respectable accuracy, with R-squared values ranging from 0.89 to 0.96. ELNET and GLM ensemble models exhibit particularly strong performance, with R-squared values of 0.96, suggesting robust predictive capabilities. Additionally, all ensemble models display minimal Root Mean Square Error (RMSE) (t/ha) and normalized RMSE (nRMSE (%)) values during calibration, indicating precise forecasting accuracy. However, during validation, the performance of ensemble models slightly diminishes, with R-squared values ranging from 0.53 to 0.77. Notably, RF ensemble models demonstrate the highest R-squared value of 0.77 during validation, suggesting relatively strong predictive accuracy. Despite this, all ensemble models experience an increase in RMSE (t/ha) and nRMSE (%) values during validation, indicating a decrease in overall predictive accuracy compared to the calibration phase. Importantly, it’s observed that individual models outperformed ensemble models in terms of both calibration and validation metrics, suggesting the potential advantages of utilizing individual modelling approaches for pearl millet yield prediction in the Jhunjhunu district.

For Jodhpur district, during the calibration phase, all ensemble models demonstrate respectable accuracy, with R-squared values ranging from 0.76 to 0.98. Notably, GLM ensemble model exhibit the highest R-squared value of 0.98, indicating robust predictive capabilities. However, Cubist and RF ensemble approaches display comparatively lower R-squared values of 0.78 and 0.76, respectively during calibration. Additionally, all ensemble models showcase minimal Root Mean Square Error (RMSE) (t/ha) and normalized RMSE (nRMSE (%)) values during calibration, suggesting precise forecasting accuracy. However, during validation, the predictive performance of the ensemble models slightly diminishes, with R-squared values ranging from 0.41 to 0.56. While RF ensemble models demonstrate the highest R-squared value during validation, GLM, ELNET and Cubist ensemble models show relatively lower R-squared values. Despite this, all ensemble models experience an increase in RMSE (t/ha) and nRMSE (%) values during validation, indicating a decrease in overall predictive accuracy compared to the calibration phase. Importantly, it’s observed that individual models outperform ensemble models, as evidenced by their superior performance in both calibration and validation phases. This emphasizes the importance of careful consideration when selecting modelling approaches for accurate pearl millet yield prediction in the Jodhpur district.

For the Nagaur district, during the calibration phase, all models demonstrate varying degrees of accuracy, with R-squared values ranging from 0.67 to 0.97. Particularly noteworthy is ELNET, which exhibits the highest R-squared value of 0.97, indicating robust predictive capabilities. However, Cubist and RF models display relatively lower R-squared values of 0.67 and 0.80, respectively during calibration. Additionally, all models showcase minimal Root Mean Square Error (RMSE) (t/ha) and normalized RMSE (nRMSE (%)) values during calibration, suggesting precise forecasting accuracy. However, during validation, the predictive performance of the models diminishes slightly, with R-squared values ranging from 0.10 to 0.62. Notably, ELNET and GLM approaches maintain relatively higher R-squared values during validation compared to Cubist and RF models. Despite this, all models experience an increase in RMSE (t/ha) and nRMSE (%) values during validation, indicating a decrease in overall predictive accuracy compared to the calibration phase. Ensemble models fail to surpass the performance of individual models for pearl millet yield forecasting over Nagaur district, thereby making individual models the preferred choice.

For the Sikar district, during the calibration phase, the ensemble models show varied performance, with R-squared values ranging from 0.49 to 0.83. Notably, ELNET demonstrates the strongest performance among the ensemble models, with an R-squared value of 0.83. However, compared to individual models, the ensemble approaches generally exhibit higher Root Mean Square Error (RMSE) (t/ha) and normalized RMSE (nRMSE) (%) values during calibration, indicating comparatively lower precision in forecasting accuracy. Conversely, during validation, the ensemble models demonstrate improvement in performance, with higher R-squared values and lower RMSE (t/ha) and nRMSE (%) values compared to the calibration phase. Despite this improvement, individual models still outperform the ensemble approaches in terms of overall predictive accuracy, as evidenced by their superior statistics matrices. Therefore, while ensemble models show a slight enhancement during validation, the suggestion leans towards utilizing individual models for more reliable pearl millet yield prediction in the Sikar district, based on their excellent performance metrics.

## Discussion

### Climatic requirements of pearl millet

Pearl millet mainly grown in agricultural settings characterized by scant rainfall, impoverished soil fertility, restricted water access and elevated temperatures, rendering it a viable option for cultivation in regions where conventional cereal crops such as rice, wheat or maize struggle to thrive [30]. Typically cultivated in areas with low rainfall ranging from 200 to 500 mm and marginal soils, pearl millet faces challenges during sowing due to inadequate soil moisture, impeding seedling emergence and crop establishment. Despite its resilience to drought stress during the vegetative growth phase, prolonged stress post-flowering can result in considerable yield reduction as the crop’s ability to recover gradually diminishes [31]. Recognized for its remarkable tolerance to high temperatures, pearl millet surpasses most other cultivated cereals, enduring temperatures as high as 42°C [32]. Temperature requisites vary depending on the variety, with an optimal range of 22–35°C for plant growth and 19–31°C for seed development [33]. Research indicates that increasing growth temperatures can decrease both individual seed weight and overall seed yield, with a more pronounced impact on yield [34]. In the semiarid regions like Rajasthan, pearl millet varieties exhibit short life cycles, typically maturing in less than 90 days to synchronize with the brief rainy season [35]. Manipulating photoperiods has been shown to influence crucial growth stages, with longer photoperiods delaying panicle initiation while potentially extending plant height and biomass. However, extending photoperiods for short-day pearl millet can also prolong the time to anthesis and alter plant morphology, adversely affecting grain yield [36]. Thus, optimal sunlight duration is imperative for timely grain filling and maximizing yield potential, particularly given the limited soil moisture available.

### Comparison of individual with ensemble models

In this investigation, a total of nine models, comprising both individual and ensemble approaches, were scrutinized for their efficacy in predicting pearl millet yield utilizing weather data across nine districts of Rajasthan. Initially, model performances were assessed employing Taylor diagrams [37] and scatter diagrams generated for each location, facilitating the identification of superior performing individual and ensemble models as detailed in the results section. However, discerning the overall best performing model across all locations remained challenging. For instance, while ensemble models exhibited subpar performance in Barmer and Nagaur, their performance ranged from satisfactory to commendable in other locations. To identify the best model, all models were ranked based on their R^2^ and nRMSE (%) values, and average ranks were computed, with lower ranks indicating superior performance [38]. Model ranking parameters were categorized into two groups namely “higher is better,” such as R^2^, and “lower is better,” such as nRMSE (%) [39]. For parameters where higher values indicate better performance, the highest values were assigned the best rank, whereas for parameters where lower values indicate better performance, the lowest values were assigned the best rank. Subsequent calculations revealed the performance order during calibration as I-GLM (1.42) >  I-XGB (1.52) >  I-RF (4.28) >  E-ELNET (4.50) >  E-GLM (5.64) >  I-ELNET (5.86) >  I-SVR (6.00) >  E-RF (7.19) >  E-Cubist (8.53), where “I” denotes individual models and “E” denotes ensemble approaches. During validation, the ranking was determined as I-SVR (3.81) >  I-ELNET (4.03) >  E-GLM (4.11) >  E-ELNET (4.14) >  E-RF (4.86) >  I-RF (5.06) >  E-Cubist (5.78) >  I-XGB (6.08) >  I-GLM (7.14). Intriguingly, while individual GLM and XGB models demonstrated superior performance during calibration, they exhibited poorer performance during validation, potentially indicating issues of data overfitting [12]. Thus, it is imperative to ascertain the overall best performing models in both calibration and validation phases. To achieve this, combined average ranks were recalculated, revealing the model performance ranking as I-XGB (3.83) >  I-GLM (4.28) >  E-ELNET (4.32) >  I-RF (4.67) >  E-GLM (4.88) >  I-SVR (4.90) >  I-ELNET (4.94) >  E-RF (6.03) >  E-Cubist (7.15). Table 4 depicts the average ranks of all models for each location.

**Table 4:** Overall average ranks of all models based on R^2^ and nRMSE (%).

Location/ Model	Individual Models					Ensemble Approaches				Best Model
	GLM	ELNET	XGB	SVR	RF	GLM	ELNET	Cubist	RF	
Alwar	5.25	4.25	3.75	6.88	7.00	4.25	2.88	5.63	5.13	E-ELNET
Barmer	1.75	5.00	4.13	4.75	4.38	5.25	4.75	7.88	7.13	I-GLM
Churu	4.63	6.25	4.88	4.38	5.63	4.88	3.88	5.00	5.50	E-ELNET
Jaipur	4.75	5.50	4.00	5.00	5.25	4.00	3.13	8.50	4.88	E-ELNET
Jalore	5.25	4.38	4.38	4.75	4.38	4.13	4.50	6.50	6.75	E-GLM
Jhunjhunu	4.63	4.38	3.50	3.50	2.50	6.88	5.63	7.88	6.13	I-RF
Jodhpur	5.25	5.13	3.00	4.00	5.00	5.50	4.50	6.50	6.13	I-XGB
Nagaur	2.63	4.50	3.38	6.25	4.38	4.00	3.13	8.50	8.25	I-GLM
Sikar	4.38	5.13	3.50	4.63	3.50	5.00	6.50	8.00	4.38	I-XGB=I-RF
Overall	4.28	4.94	3.83	4.90	4.67	4.88	4.32	7.15	6.03	I-XGB

In Alwar, Churu, and Jaipur districts, the ensemble ELNET approach emerged as the top-performing model for predicting pearl millet yield, whereas in Jalore district, the ensemble GLM model exhibited the highest efficacy. These findings align with previous research by [40,41], which also observed the superiority of ensemble approaches over individual models. Conversely, the individual GLM model demonstrated superior performance in Barmer and Nagaur districts. Additionally, individual RF and XGB models outperformed others in Jhunjhunu and Jodhpur districts, respectively, while for Sikar district, both individual XGB and RF models yielded identical scores for pearl millet yield forecasting. This pattern of individual models outperforming ensemble approaches is consistent with findings by [15,42]. Similarly, findings of [43] suggest that ensemble models generally performed well, they did not consistently outperform their individual counterparts, supporting the results of this study. Among the nine locations examined, ensemble approaches outperformed individual models four times, while individual models proved superior in five instances for predicting pearl millet yield. In the overall ranking, individual XGB and GLM models emerged as the top performers. However, considering the potential issue of data overfitting, the ensemble ELNET approach is recommended, followed by the individual RF model, for accurate prediction of pearl millet yield.

Although the models demonstrate high accuracy in predicting pearl millet yield, it is essential to acknowledge that even small forecast errors can have significant consequences in operational settings. For example, inaccurate yield predictions may lead to suboptimal decisions regarding irrigation scheduling, input planning (such as fertilizer or pesticide use), and yield estimation for market supply. These errors, if unaccounted for, can result in either resource overuse or shortages, ultimately affecting farm profitability and food security.

To mitigate the risks associated with forecast errors, we propose a cautious approach where machine learning models are combined with expert knowledge and historical data to enhance decision-making. By integrating human expertise and additional contextual information, stakeholders can account for factors that the models might overlook, such as sudden weather anomalies or socio-economic variables that influence farming practices. This hybrid approach offers a more robust and reliable system for agricultural planning, allowing for better resource allocation and reducing the negative impacts of potential forecasting errors.

## Conclusion

Through rigorous evaluation using Taylor diagrams and scatter diagrams, superior performing models were identified at each location. Despite variations in performance across districts, ensemble ELNET emerged as the top-performing model in Alwar, Churu, and Jaipur, while ensemble GLM excelled in Jalore district. These findings support prior research indicating the superiority of ensemble approaches over individual models in certain contexts. However, notable exceptions were observed, with individual GLM demonstrating superior performance in Barmer and Nagaur districts and individual RF and XGB models outperforming others in Jhunjhunu and Jodhpur districts, respectively. These performances underscore the importance of tailored model selection based on specific geographic and environmental conditions. While ensemble models generally performed well in the study, they did not consistently outperform individual models, aligning with previous studies. There are several potential reasons why the ensemble models in this study may have underperformed compared to individual models in certain districts. One significant factor could be data overfitting, where the ensemble models, due to their complexity, may have fit too closely to the training data, capturing noise rather than meaningful patterns. This can lead to reduced performance during validation or testing when new, unseen data is introduced. Additionally, model complexity in ensemble methods can sometimes lead to difficulties in capturing the specific, subtle relationships between the predictors and yield that individual models may handle more effectively. Ensembles often combine multiple algorithms, and this complexity can lead to issues such as poor generalization, especially when the data quality or quantity is limited. Moreover, interpretability issues arise as ensemble models can obscure the underlying relationships between variables, making it challenging to understand the key drivers of yield variability, which individual models might more transparently reveal. These factors combined could explain the unexpected performance outcomes in this study.

Overall, considering the potential issue of data overfitting, the ensemble ELNET approach is recommended for accurate prediction of pearl millet yield, followed by the individual RF model. This study contributes valuable insights into the optimization of machine learning approaches for agricultural yield forecasting in diverse agro-climatic regions.

## Limitations

One limitation of this study pertains to the availability of long-term yield data, which could further validate the efficacy of individual and ensemble approaches over extended periods. While the current analysis provides valuable insights into model performance based on existing data, the lack of long-term yield datasets limits the assessment of model robustness and predictive accuracy over multiple growing seasons. Another potential limitation of this study could be the reliance on weather data alone for pearl millet yield prediction, without considering other relevant factors such as soil quality, pest infestations, or agronomic practices. While weather variables play a crucial role in crop growth and development, omitting other influential factors may restrict the comprehensive understanding of yield variability and limit the accuracy of predictive models.

Another significant constraint is the overfitting observed in the XGB and GLM models. During calibration, these models demonstrated excellent performance, but their predictive accuracy declined sharply during validation, indicating that they were too closely fitted to the training data and failed to generalize well to new data. This overfitting issue suggests that the use of XGB and GLM models in isolation should be approached with caution, as they may not provide reliable predictions in practical, real-world applications.

## Future prospect

The findings of this study pave the way for several promising avenues of future research in the field of pearl millet yield forecasting. First and foremost, further investigations could delve deeper into refining the existing predictive models by incorporating additional data sources and variables, such as soil quality, agronomic practices, and pest management strategies, to enhance the accuracy and robustness of the models. Additionally, longitudinal studies involving multi-year data collection would provide valuable insights into the long-term performance and reliability of both individual and ensemble approaches across diverse climatic and agronomic conditions. Furthermore, exploring the applicability of advanced machine learning techniques, such as deep learning algorithms, and integrating remote sensing data for real-time monitoring and prediction of pearl millet yield could represent promising avenues for future research.
